# Exploring Potential Epigenetic Biomarkers for Colorectal Cancer Metastasis

**DOI:** 10.3390/ijms25020874

**Published:** 2024-01-10

**Authors:** Priyadarshana Ajithkumar, Sai Shyam Vasantharajan, Sharon Pattison, John L. McCall, Euan J. Rodger, Aniruddha Chatterjee

**Affiliations:** 1 Department of Pathology, Dunedin School of Medicine, University of Otago, Dunedin 9016, New Zealand; ajipr506@student.otago.ac.nz (P.A.);; 2 Department of Medicine, Dunedin School of Medicine, University of Otago, Dunedin 9016, New Zealand; 3 Department of Surgical Sciences, Dunedin School of Medicine, University of Otago, Dunedin 9016, New Zealand; 4 School of Health Sciences and Technology, UPES University, Dehradun 248007, India

**Keywords:** colorectal cancer, DNA methylation, epigenetics, metastasis, lymph node, liver, histone modification, biomarkers

## Abstract

Metastatic progression is a complex, multistep process and the leading cause of cancer mortality. There is growing evidence that emphasises the significance of epigenetic modification, specifically DNA methylation and histone modifications, in influencing colorectal (CRC) metastasis. Epigenetic modifications influence the expression of genes involved in various cellular processes, including the pathways associated with metastasis. These modifications could contribute to metastatic progression by enhancing oncogenes and silencing tumour suppressor genes. Moreover, specific epigenetic alterations enable cancer cells to acquire invasive and metastatic characteristics by altering cell adhesion, migration, and invasion-related pathways. Exploring the involvement of DNA methylation and histone modification is crucial for identifying biomarkers that impact cancer prediction for metastasis in CRC. This review provides a summary of the potential epigenetic biomarkers associated with metastasis in CRC, particularly DNA methylation and histone modifications, and examines the pathways associated with these biomarkers.

## 1. Introduction

Colorectal cancer (CRC) is the third most prevalent cancer and the second leading cause of cancer-related mortality worldwide [1]. It is the second most frequent cancer among women and the third among men [2]. Approximately 1.8 million people are expected to develop CRC annually, and around half of these patients are likely to die of the disease [3]. The incidence rates of CRC are among the highest in Australia and New Zealand [4]. While advancements in CRC management have led to improved outcomes, the prognosis varies significantly across different stages of the disease [5,6,7,8]. The American Joint Committee on Cancer (AJCC, 8th edition) TNM staging system is a widely accepted classification system of tumours [9]. The TNM staging system categorises tumours based on the primary tumour size (T classification), lymph node involvement (N classification), and distant metastasis (M classification), ultimately determining an overall stage group from I to IV [9]. Based on TNM classification, stage I and II signify local disease only, including the absence of any metastasis; stage III is defined by the presence of lymph node metastases, while stage IV indicates the distant spreading of cancer [9]. The stage of CRC correlates with the five-year survival rate, indicating that early stages (stage I and stage II) exhibit higher survival rates compared to advanced stages (stage III and stage IV). For instance, it was reported that the five-year survival rate was approximately 90% for stage I CRC, ranging from 63% to 87% for stage II and from 53% to 89% for stage III [7]. However, patients diagnosed with distant metastases (stage IV) have been reported to have a considerably lower five-year survival rate, as low as 14% [8]. The presence of distant metastases, which define stage IV disease, substantially influences the outcome and plays a pivotal role in worsening the prognosis in CRC patients [10].

Metastasis is the primary contributor to high mortality rates in CRC due to the high burden caused by various impacts on organ function due to distant metastasis [11,12]. Furthermore, around 25% of individuals have distant metastasis at the time of diagnosis [11]. Common sites for distant CRC metastasis are the liver, lungs, brain, and bone [13,14,15,16,17]. Approximately half of individuals diagnosed with CRC eventually develop liver metastasis, and the liver is the most frequently identified site of metastatic disease [12,13]. The lung is the second most frequently involved metastatic site, observed in around 10–15% of all metastasis cases, followed by the bone (detected in 1.2–12% CRC patients) and brain (incidence between 0.3 and 9%) [15,16,17]. Despite all metastases resulting in the poor median survival of patients with CRC, this varies by metastasis site as follows: liver (5 to 20 months), lung (5.9 to 31.2 months), bone (5 to 21 months), and brain (1 to 2 months) [17,18,19,20]. The five-year survival rates for these metastases are 15.99% for the liver, 16.70% for the lung, 5.51% for the brain, and less than 5% for bone metastasis [17,19]. These observations mentioned above show the prognosis of metastasis in CRC, particularly its spread to vital organs. Identifying metastases plays a pivotal role in determining the disease stage and guiding the treatment intent [9,21].

The process of accurately detecting CRC metastasis is often performed through the use of imaging technologies [22]. Various imaging modalities are currently used in clinical practice to stage CRCs, each offering distinct strengths and limitations. Computed tomography (CT) scans can detect metastasis to the regional lymph nodes and other organs [23,24,25,26]. A contrast-enhanced CT scan is the standard imaging technique used for pre-operative local staging in colon cancer cases [27]. The limitations of CT scans include poor sensitivity to small-sized primary cancers and an inability to definitively distinguish metastatic nodes [26,28]. The accuracy of CT scans to detect lymph node metastasis is around 61–67% [29]. CT can also be combined with fluorodeoxyglucose positron emission tomography (FDG-PET) to detect otherwise occult metastases that conventional imaging methods might fail to detect [30,31]. The limitation of FDG-PET is that non-malignant conditions such as inflammation, fibrosis, and oedema can have increased FDG avidity, resulting in false positives [23]. Furthermore, FDG-PET may miss the detection of lesions smaller than 1 cm, and patients with mucinous CRC may yield false-negative results due to the low FDG uptake associated with the mucinous component [32,33,34].

Magnetic resonance imaging (MRI) is commonly used for staging rectal cancer and detecting metastases in the liver, brain, and bones in cases of CRC [11,35,36,37]. MRI’s effectiveness is particularly distinguished by its high detection rate, which is capable of identifying lesions smaller than 10 mm [22] and could be missed by FDG-PET. However, one of the limitations of MRI is that it is unreliable in detecting lymph node metastasis with a pooled sensitivity of 0.77 and specificity of 0.71 [37,38]. Imaging-based technologies require experienced physicians to interpret these findings, which interpretation is subjective and can be varied, running the risk of under or over-calling the extent of the disease [39]. At present, the lack of a reliable method to identify metastasis without depending on imaging highlights the current challenges in the early detection and prediction of metastatic lesions in CRC [40,41], emphasizing the need for alternative approaches. The detection of molecular-based markers can offer a potential means for predicting metastasis [42].

Genetic changes and mutations that occur in oncogenes and tumour suppressor genes (TSGs) were previously regarded to be driving events for tumour initiation and progression [43]. The key genetic mutations associated with CRC metastasis are *RAS*, *BRAF*, *PIK3CA* and *TP53* [44]. An interplay exists between genetic mutations and epigenetic changes. Dysregulation or mutations in epigenetic enzymes can induce epigenetic modifications, thereby influencing gene expression and protein stability, ultimately promoting the metastatic process [45,46]. More recently, evidence of epigenetic changes in driving cancer formation and metastasis is emerging [47,48]. Furthermore, epigenetic modifications have been recognised as important events in CRC carcinogenesis and metastasis [42,43,49]. For example, DNA methylation is an important factor in contributing to metastasis in various cancers, including CRC [43,50,51,52]. Histone modifications are another type of epigenetic modification and are important in the development, advancement, metastasis, and resistance to drugs in CRC [53].

Numerous DNA methylation and histone biomarkers linked to CRC metastasis have been recognised. The identification of DNA methylation biomarkers involves the use of various methodologies, and their choice depends on whether global or regional methylation analysis is performed [54]. This includes a methylation-specific PCR (MSP), quantitative methylation-specific PCR (Q-MSP), microarray analysis, pyrosequencing, and reduced representation bisulfite sequencing (RRBS) [42,55,56,57,58,59,60,61,62]. MSP is a PCR-based technique that amplifies specific DNA regions after bisulfite conversion, identifying the methylation status at precise loci using primers targeting methylated or unmethylated sequences [63]. Q-MSP quantifies DNA methylation levels at specific CpG sites through a real-time PCR using methylation-specific primers and fluorescent probes or dyes emitting signals during amplification [63]. Illumina (San Diego, CA, USA) has developed various human microarray platforms for DNA methylation analysis [64]. These include HumanMethylation27 BeadChip (>27,000 CpGs), HumanMethylation450 BeadChip (>450,000 CpGs), HumanMethylationEPIC BeadChip (>850,000 CpGs), and HumanMethylationEPIC version 2.0 (>900,000 CpGs) [64]. Pyrosequencing is a quantitative method that is used to determine the percentage of methylation at specific CpG sites [65]. RRBS utilises the *MspI* enzyme to digest DNA prior to bisulfite conversion, which targets CG-rich regions and sequences ~2.5% of the human genome [66,67,68]. The detection of histone biomarkers is achieved through techniques such as immunohistochemistry (IHC), Western blot assays, immunoprecipitation, and quantitative PCR (qPCR) [69,70,71,72,73,74,75,76,77,78,79,80,81,82,83,84,85,86,87,88,89]. The IHC method is used to visualise and quantify specific histone modifications in tissue samples using antibodies that bind to modified histones [90,91,92]. Western blot assays are used to examine histone modifications by separating proteins based on their size and charge [93,94]. DNA obtained via immunoprecipitation is used in subsequent qPCR to identify the occurrence of particular histone marks at specific genomic locations [95]. In this review, we provide an overview of the DNA methylation and histone biomarkers that have been identified in relation to the lymph node, liver, lung, and other distant metastases in CRC. We explore findings derived from tissue samples, as well as the results obtained from in vitro and in vivo experiments.

## 2. DNA Methylation Biomarkers for CRC Metastasis

DNA methylation is a mechanism contributing to tumour initiation, growth, advancement, progression, recurrence, and metastasis [96,97]. DNA methylation involves the attachment of a methyl group to the C5 position of cytosine–guanine dinucleotides (CpG) [96,98,99]. CpG-rich regions are known as CpG islands, defined as regions with a GC content greater than 50%, a CpG ratio higher than 60%, and a minimum length of 200 base pairs [98,99]. Hypermethylation (the gain of methylation) in the CpG-rich promoter regions causes the silencing of transcriptional genes in CRC [97]. Genome-wide methylation sequencing via the methyl-seq of paired tissues which include normal adjacent, primary tumour and lymph node metastasis from three CRC patients, showed lower CpG island hypermethylation in lymph node metastasis when compared to primary tumours (*p*-value < 0.001) and higher hypermethylation than normal adjacent tumours (*p*-value < 0.001) [100]. The results suggest that while there might be changes in methylation levels during metastasis, there is some conservation of methylation patterns in CpG islands during the progression of CRC from the primary tumor to lymph node metastasis. A large sample size can increase the accuracy of the results. Hypomethylation (the loss of DNA methylation) occurs most commonly in open sea regions of the genome and is associated with chromosomal instability (CIN), gene activation, and the loss of imprinting in CRC [97].

### 2.1. DNA Hypermethylation of Tumour Suppressor Genes as a Biomarker for Lymph Node Metastasis

TSGs tightly regulate cell division in normal cells, inhibiting tumour formation [101]. However, an aberrant increase in TSG promoter methylation results in the silencing of TSGs [98]. TSG promoter hypermethylation is widely observed in multiple cancer types, including CRC, hepatocellular carcinoma, epithelial ovarian cancer, non-small cell lung cancer, and prostate cancer [102,103,104,105]. In CRC primary tumours, the hypermethylation of bone morphogenetic protein 2 (*BMP2*), cyclin-dependent kinase inhibitor 2A (*CDKN2A*), and family with sequence similarity 134 member B (*FAM134B*) was associated with lymph node metastases [55,56,57]. In a cohort of 498 CRC patients across stages I, II, and III (TNM 7th edition), *BMP2* hypermethylation was detected in 60% of cases [55]. In this patient subset, a distinct trend emerged, indicating alterations in *BMP2* methylation across the different disease stages. A higher proportion of *BMP2* methylation was observed in stage I/II (54%, 163 patients) compared to stage III (46%, 139 patients) [55]. Indeed, *BMP2* hypermethylation was associated with patients with lymph node metastases (*p*-value = 0.012) and stage III disease (*p*-value = 0.010) [55]. Furthermore, the subset of patients with hypermethylated *BMP2* had a poorer prognosis, particularly left-sided stage III patients (*p*-value = 0.031) [55]. *BMP2* is a member of the transforming growth factor (TGF)-β superfamily that acts via the Smad signalling pathway and has a critical role in regulating cell proliferation, differentiation, and apoptosis [55]. The downregulation of *BMP2* was observed previously in CRC [106], possibly as a result of DNA promoter methylation, but it has not yet been established whether this has a causal role in metastasis. Although not included in this study [55], the methylation status of *BMP2* in stage IV patients is of interest to provide a better understanding of its role in distant metastasis and other organs.

Lymphovascular invasion (LVI) is the presence of cancer cells in lymph or blood vessels in the primary tumour, demonstrating the ability of cancer cells to intravasate, which is crucial for metastasis [107]. LVI correlates with an elevated risk of lymph node metastases and tumour invasion to the extramural veins, which is subsequently linked to distant metastases [57]. CRC patients with LVI detected in their primary tumours tend to have a higher likelihood of lymph node metastasis compared to those without LVI [108]. TSGs such as *CDKN2A* and *FAM134B* were found to be hypermethylated in CRC patients with lymph node metastasis and in patients with LVI [57,58], highlighting the potential involvement of multiple hypermethylated TSGs in the metastatic cascade and the invasive traits of CRC. *FAM134B* genes function to regulate Endoplasmic Reticulum (ER) turnover through autophagy (the removal of cellular waste, including damaged proteins and organelles) [57,109]. A comprehensive analysis showed a disparity in *FAM134B* methylation levels among patients with late (stage III or IV) and early stages (stage I or II). Late-stage CRC patients had a higher occurrence of *FAM134B* methylation (76%) in comparison to the early stage (33%) [57]. There was a higher prevalence of *FAM134B* methylation among patients with lymph node metastasis (75% of patients) and those exhibiting LVI (68% of LVI-positive patients) when compared to patients without any lymph node metastasis or LVI-negative patients [57]. *FAM134B* methylation could have a role in various stages of cancer progression, and it can potentially affect the likelihood of lymph node metastasis by facilitating tumour invasion through lymphovascular structures. It could also potentially influence metastatic pathways and collectively drive the progression of cancer to metastasis.

Similarly, an alteration in the methylation status of another TSG *CDKN2A* has been increasingly recognised as a pivotal factor in CRC lymph node metastasis. A meta-analysis involving 3440 CRC patients encompassing stage I to stage IV tumours revealed that individuals with LVI or lymph node metastasis had a higher likelihood (1.68 times) of exhibiting *CDKN2A* hypermethylation than patients without LVI or lymph node metastasis (positive vs. negative: an odds rations of 1.68) [58]. Furthermore, individuals with hypermethylated *CDKN2A* have a 1.65 times higher risk of experiencing decreased OS compared to those without hypermethylation (95% CI 1.29–2.11). No significant associations were observed between *CDKN2A* hypermethylation and other clinicopathological features [58], indicating the relevance of *CDKN2A* hypermethylation, specifically to lymph node metastasis and survival outcomes. The *CDKN2A* gene is important for cell differentiation, cellular senescence, and death [110]. An abnormal modification in *CDKN2A* could result in the silencing of the *CDKN2A* gene, impacting its tumour-suppressive functions [58].

Although a link was found between *CDKN2A* methylation and CRC’s spread to the lymph nodes [56,58], findings of this association are not consistent in the literature. For instance, an analysis of sporadic adenocarcinomatous CRC tissues from both the colon and rectum revealed a higher prevalence of *CDKN2A* methylation in stage II patients (*p*-value = 0.012) and those without lymph node metastasis (*p*-value = 0.011). Furthermore, this study also revealed that *CDKN2A* methylation was less frequent in stage III (*p*-value = 0.016) [59]. The discordant results in the literature describing the relationship between *CDKN2A* gene methylation and lymph node metastasis indicate that other variables such as sample variability, tumour heterogeneity, methodological differences in analysis, potential environmental influences, and variations in statistical approaches may need consideration and further analyses are required. It is also plausible that *CDKN2A* DNA methylation could be variably altered in different stages of metastasis. However, there is relatively little data available on *CDKN2A* methylation levels at different stages of CRC. Despite research showing the roles of TSGs like *CDKN2A* and *FAM134B* in CRC lymph node metastasis [57,58], the studies that explore underlying mechanisms associated with lymph node metastasis and invasion into LVI are limited.

### 2.2. Hypomethylation of the Enzyme-Encoding Gene as a Biomarker for Lymph Node Metastasis

Enzymes are catalysts that accelerate chemical reactions within living organisms [111]. The enzyme glucosaminyl (N-acetyl) transferase 2 (*GCNT2*) plays a crucial role in glycosylation and the synthesis of I-branched glycans [112,113]. GCNT2/I-branched glycans are important for cancer progression, adhesive, migration, proliferation, signalling, and metastasis [114]. The hypomethylation of *GCNT2* has emerged as a potential biomarker linked to lymph node metastasis in CRC [60]. Studies have reported that the diagnostic accuracy for predicting lymph node metastasis via *GCNT2* hypomethylation in primary tumours was 86.2% which is higher than the diagnostic accuracies of CT (59.38%) and MRI (84%) methods used to predict lymph node metastasis in CRC [26,60,115]. Interestingly, the level of *GCNT2* hypomethylation was found to be similar between the tumour tissues and their corresponding normal tissues, suggesting the potential use of *GCNT2* hypomethylation in normal mucosa tissue to predict lymph node metastasis in CRC patients [60]. Utilising normal mucosa tissue for predicting lymph node metastasis offers a potentially earlier and more accessible method for predicting lymph node metastasis in CRC patients. However, the study by Nakamura et al. [60] did not report specific diagnostic accuracy values for predicting lymph node metastasis in normal tissue. Since quantitative diagnostic accuracy values in normal tissue were not provided, it is difficult to compare the diagnostic accuracy between normal mucosa and primary tumours or to compare it against other imaging systems for predicting lymph node metastasis.

### 2.3. DNA Hyper and Hypomethylation as an Early Event to Predict Liver Metastasis

Colorectal liver metastases (CLMs) are present in about 20% to 25% of CRC patients at initial diagnosis, with 40% to 50% of patients developing liver metastasis post-primary resection [116]. The liver is the most frequent site for CRC metastasis because of intestinal mesenteric drainage to the hepatic portal veins [117]. Changes in DNA methylation patterns, including both hypermethylation and hypomethylation events, occur in the early stage of CRC (before the tumour cells have metastasised to the liver) [61,117]. To identify differentially methylated regions (DMRs) linked to liver metastasis prediction in CRC, 59 primary tumour samples were analysed, among which 22 patients developed liver metastasis, while 37 did not during the follow-up period [117]. The Least Absolute Shrinkage and Selection Operator (LASSO) regression model was applied, revealing 23 DMRs linked to an increased risk of liver metastasis. These identified regions hold promise as predictive markers in early-stage CRC patients [117]. Moreover, Leave-One-Out Cross-Validation (LOOCV) was performed to validate the predictive model, and the results showed an area under the curve of 0.701 (sensitivity = 72.7%, specificity = 70.3%) [117]. These results are promising for developing markers to predict the likelihood of liver metastasis. While LOOCV results showed predictive capability, there is potential for further improvement to achieve higher sensitivity and specificity. Increasing the number of patient data to construct this model could increase the sensitivity and specificity. For example, a study used a logistic regression model and data (histological images obtained from primary tumour samples) from 300 patients diagnosed with stage I, stage II, or stage III CRC to predict lymph node metastasis in CRC. The model exhibited a sensitivity of 0.81, a specificity of 0.87, and an area under the curve of 0.91 [118]. The higher level of accuracy observed in the model might be due to the fact that it was built using a larger dataset consisting of 300 patients.

An integrated omics approach that combines methylation analysis with transcriptome profiling from paired primary tumour and liver metastatic tissues presents a powerful strategy to identify epigenetic markers linked to the prediction of liver metastasis. This approach enabled the simultaneous analysis of DNA methylation patterns and gene expression changes, which provided a multi-dimensional view of the molecular and functional alterations occurring during CRC progression and metastasis to the liver. Using RRBS, a panel of 244 differentially methylated CpGs (DMCs) (68 hypomethylated and 176 hypermethylated) were identified in liver metastatic tissues (*n* = 10) in comparison to paired (i.e., from the same patients) primary CRC tissues (*n* = 10) [42,62]. The majority of the 244 DMCs exhibited hypomethylation in the normal colon and primary CRC, which eventually changed to hypermethylation in liver metastasis. The integration of methylation data (165 DMCs—genes or regulatory regions) with matched transcriptome data showed that the methylation changes of 21 DMCs were highly correlated with alterations in the expression of 20 protein-coding genes linked to those 21 DMCs (a Spearman or Pearson correlation coefficient < 0.4, *p*-value < 0.05) [42,62]. These 21 DMCs could possibly be epigenetic drivers of CRC metastasis [42]. The study also demonstrated that the methylation signature of metastasis is independent of the driver mutation status of the patients, suggesting that the mutation agnostic methylation signature could be developed or metastasis. However, it is necessary to perform such studies in larger patient cohorts. These studies help to understand the molecular changes in DNA methylation and gene expression that occur during the metastatic process, which can provide insights into the mechanisms underlying metastasis. Currently, this is the only study that has investigated methylation patterns and transcriptome profiles in paired samples of CRC primary tumours and liver metastasis using sequencing-based analyses. Such integrated analyses should be performed in larger cohorts and could strengthen the reliability and accuracy of biomarkers, ensuring their clinical applicability.

Understanding DNA methylation alterations in primary CRC could offer insights into its role as an early event and its impact on liver metastasis. When comparing primary CRCs with and without liver metastasis, no substantial differences were noted in the methylation status of ten genes [61]. Methylation levels of *p14*, a tissue inhibitor of metalloproteinase 3 (*TIMP3*), and hyperplastic polyposis 1 (*HPP1*) exhibited a gradual decrease from the absence to the presence of liver metastasis in primary tumours. [61]. Moreover, out of these 21 genes, only methylguanine methyltransferase (*MGMT*) exhibited higher methylation in liver metastasis compared to the primary tumour [61]. The other 20 genes did not display any significant differences [61]. The results were further validated using bisulfite pyrosequencing in 12 paired samples, which indicated that most of the observed increases were inconsistent and the variations could be due to methylation density rather than frequency [61]. The validation performed using pyrosequencing showed that most of the selected genes had a similarity in the methylation frequency between primary tumours and corresponding liver metastasis [61]. The authors concluded that DNA methylation is an early event, and methylation changes occur in tumour cells before cells progress to liver metastasis [61].

### 2.4. The Role of DNA Hypermethylation in Distant Metastasis

**Figure 1:**
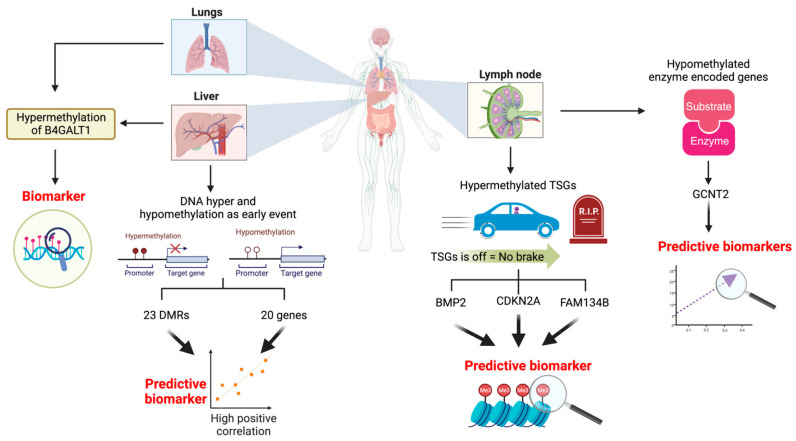
Schematic diagram of DNA methylation changes in genes linked to CRC metastasis. The hypermethylation of TSGs, including *BMP2*, *CDKN2A*, and *FAM134B*, could potentially serve as prognostic indicators for CRC lymph node metastasis. Conversely, the hypomethylation of genes encoding GCNT2 enzymes may function as predictive markers for lymph node metastasis. DNA hypermethylation and hypomethylation in 23 DMRs and 20 other genes might offer predictive insights for CRC liver metastasis. *B4GALT2* hypermethylation may have the potential to be utilized as a diagnostic indicator for lung and liver metastasis. These include bone morphogenetic protein 2 (*BMP2*), cyclin-dependent kinase inhibitor 2A (*CDKN2A*), a family with sequence similarity 134 member B (*FAM134B*), glucosaminyl (N-acetyl) transferase 2, the I-branching enzyme (*GCNT2*) and beta-1,4-galactosyltransferase 2 (*B4GALT2*). The lollipop structures displaying brown-coloured circles represent methylated CpG sites, while the white circles indicate unmethylated CpG sites. The arrow marked with a red-coloured cross signifies blocked transcription, while the arrow without a cross indicates active transcription.

Despite extensive research on DNA methylation patterns in CRC metastasis, information on specific DNA methylation biomarkers for metastatic sites such as the lung, bone, brain, and other organs remains limited. Specific genes, such as beta-1,4-galactosyltransferase (*B4GALT1*), have been identified as hypermethylated in CRC liver and lung metastase lesions [119]. No studies to date have successfully identified DNA methylation biomarkers that are specific to the lung, bone, brain, or other organs of metastasis. Given the infrequency of bone and brain metastasis, along with the poor prognosis associated with these metastatic sites in CRC patients [11,120], the identification of biomarkers is challenging due to the low number of patients and the difficulty in obtaining tissue samples for analysis. The literature described in this section on methylation biomarkers for CRC metastasis is summarised schematically in Figure 1.

## 3. Histone Modifications as Potential Biomarkers for CRC Metastasis

Histones are proteins that assist in organising genomes into nucleosomes, and each nucleosome consists of eight histone proteins [121]. These proteins help with DNA condensation, organisation, and regulation of gene expression [121]. Histones undergo various modifications, such as methylation, acetylation, and phosphorylation, that prompt chromatin remodelling, which alters the histone structure [122,123]. The alteration of the histone structure is known to play a regulatory role in gene transcription, chromatin structure, replication, DNA repair, and recombination [122,123]. The aberrant regulation of histone modification can initiate tumour formation, progression, and metastasis by influencing the expression of TSGs and oncogenes [122]. The following section focuses on acetylation, methylation, and ubiquitination, as these were found to be the most researched histone modifications in CRC metastasis to date.

### 3.1. The Role of Histone Deacetylase Enzymes in CRC Metastasis

Histone deacetylases (HDACs) are responsible for eliminating acetyl groups from lysine residues on histones and regulate transcription, apoptosis, stress responses, DNA repair, cell cycle, and genomic stability [124,125]. In tumour cells, HDACs induce modifications in the nucleosome structure, consequently altering gene transcription and promoting the expression of genes associated with cell proliferation, migration and metastasis [69]. The HDAC enzyme family is classified into the following four major classes: class I (consisting of HDAC1, HDAC2, HDAC3, and HDAC8), class II (HDAC4, HDAC5, HDAC6, HDAC7, HDAC9, and HDAC10), class III (Sirtuin 1 to Sirtuin 7) and class IV (HDAC11) [126]. Accumulating evidence has revealed the involvement of specific HDAC enzymes, including HDAC1, HDAC2, HDAC6, and Sirtuin 2 (SIRT2), in CRC metastasis [69,70,71,72,73,74,75,76,77]. Cancer cells need to acquire characteristics such as proliferation, migration, and invasion in order to metastasise successfully [127]. *HDAC1* is shown to play a role in the acquisition of these characteristics in CRC cells [71]. For instance, a study explored the effect of *HDAC1* expression status on the proliferation, migration, and invasion of SW48 and LOVO human CRC cell lines using the transwell assay [71]. The overexpression of *HDAC1* led to an increase in the proliferation, migration, and invasion of CRC cells, while the silencing of *HDAC1* had the opposite effect on these characteristics [71]. However, the study by Chen et al. [71] did not perform in vivo experiments to demonstrate the development of these characteristics that lead to metastasis. Hence, in vivo experiments are required to better understand the role of *HDAC1* in CRC metastasis.

#### 3.1.1. Histone Deacetylases Influence the Progression of Epithelial-to-Mesenchymal Transition

Epithelial-to-mesenchymal transition (EMT) is a cellular process where cells shift from an epithelial phenotype to a more motile, mesenchymal state [128]. EMT is a significant contributor to metastasis, granting metastatic cells the ability to migrate and invade, resist apoptosis, and evade immune detection [129]. HDACs modulate EMT, a key factor contributing to the metastasis of CRC [72,78]. Wang et al. [78] illustrated the formation of a complex comprising HDAC1, HDAC2, the enhancer of Zeste Homolog 2 (EZH2), and Snail. This complex functions to inactivate the Disabled Homolog 2-Interacting Protein (*DAB2IP*) in CRC cell lines [78]. *DAB2IP* inhibits proliferation, invasion, EMT, tumour growth, and metastasis in CRC cell lines [78]. Similarly, another complex involving HDAC2, HDAC1, and EZH2 relies on long non-coding RNA (lncRNA) *ENSG00000274093.1* to facilitate their interaction [72]. This interaction promotes traits such as EMT, migration, and invasion in CRC cell lines [72]. The expression of lncRNA *ENSG00000274093.1* was studied in normal tissue, and primary tissues were obtained from patients with and without liver metastasis [72]. It was found that there was a gradual increase in the expression from normal tissue to primary tissue without liver metastasis to the primary tumour with liver metastasis (*p*-value < 0.01) [72]. The association observed between this lncRNA and the HDAC2/HDAC1/EZH2 complex suggests a potential link to CRC liver metastasis.

#### 3.1.2. Involvement of Histone Deacetylases in CRC Liver Metastasis

HDACs such as *HDAC2* and *SIRT2* have been shown to be involved in the progression of CRC liver metastasis [72,77]. Exploring *HDAC2* expression in primary tumours obtained from patients with and without liver metastasis revealed that *HDAC2* was highly expressed in patients with liver metastasis compared to those without liver metastasis (*p*-value < 0.01) [72]. Moreover, survival analysis indicated that a high *HDAC2* expression was significantly associated with poorer outcomes (Hazard Ratio = 2.283, *p*-value = 0.031) [72]. Comparing *HDAC2* expression in tumours from patients with and without liver metastasis and a survival analysis association suggested *HDAC2*’s potential role in CRC liver metastasis. In the context of *SIRT2*, it was observed that *SIRT2* was significantly downregulated in primary tumours obtained from CRC patients with liver metastasis (all six patients had low expression) compared to corresponding normal tissues [77]. Further, the role of *SIRT2* in the acquisition of migration and invasive properties via CRC cells was investigated in HCT116 cell lines using cell invasion and migration assays [77]. The results show that high expressions of *SIRT2* inhibited migration and invasion [77]. However, the study by Wang et al. [77] needs to be performed on in vivo models to demonstrate that the increase in the migratory and invasive potential of CRC cells, mediated by *SIRT2*, contributes to liver metastasis.

### 3.2. Histone Methyltransferases as Potential Oncogenes in CRC Liver Metastasis 

Oncogenes play a pivotal role in inducing normal cells to adopt a neoplastic phenotype. Certain histone methyltransferases (HMTs) have been identified as oncogenes in CRC [79,80,130,131]. HMTs are enzymes that methylate lysine or arginine residues of histones and influence transcriptional activity in normal cells [132,133]. Abnormal changes in HMT expression have been linked to CRC development, progression, and metastasis [80,132]. Evidently, various HMTs, including the Suppressor of Variegation 3–9 Homolog 2 (*SUV39H2*) [79] and SET Domain Bifurcated Histone Lysine Methyltransferase 1 (*SETDB1*) [80] were associated with CRC liver metastasis. *SUV39H2* influences various critical stages of the metastatic cascade, including proliferation, migration, and invasion in vitro [79]. In vivo experiments revealed an association with liver metastasis due to the increased *SUV39H2* expression [79]. *SUV39H2* facilitates CRC metastasis by binding to the SLIT guidance ligand 1 (SLIT1) promoter, inducing histone H3 lysine 9 (H3K9) tri-methylation and resulting in the downregulation of *SLIT1* [79]. *SETDB1* is another HMT linked to CRC metastasis [80]. *SETDB1* displayed an elevated expression in liver tissue obtained from colon cancer patients compared to primary and normal mucosa tissues, with a *p*-value of <0.001 [80]. However, the study did not report further analysis on liver metastasis, such as *SETDB1*-based in vivo experiments, pathway analysis, or multi-omics analysis, to further comprehend the function of *SETDB1* in colon liver metastasis. Utilising an in vivo model can help to investigate the metastatic process at each stage of metastasis, while a multi-omics approach allows for the identification of significant molecular changes associated with metastasis, including genomic and cellular heterogeneity [134,135].

### 3.3. Lysine-Specific Histone Demethylases Promote CRC Metastasis

Histone Demethylases (HDMs) remove the methyl group from histones and regulate gene expression [136,137]. HDMs are classified into the following two groups: Lysine-Specific Demethylases (LSDs) and Jumonji Domain-Containing (JMJD) Demethylases [138,139]. The aberrant expression of HDMs has been identified in multiple cancers, including CRC [81,82,83,140,141,142,143,144]. Among the dysregulated LSDs, Lysine Demethylase 3A (*KDM3A*) and *LSD1* showed elevated expression levels in metastatic lesion tissue compared to paired CRC primary tissue (*p*-value < 0.001) and distant metastasis in primary tissue (*p*-value < 0.05), respectively [81,82,83]. The high expression of *KDM3A* and *LSD1* expressions were linked to metastatic properties involving the promotion of invasion and proliferation in CRC cell lines [81,82,83,144]. Moreover, *KDM3A* expression has been correlated with stage III-IV (*p*-value = 0.002), N (*p*-value = 0.001), and M classification (*p*-value = 0.001) based on the TNM classification system [81]. The upregulation of *KDM3A* and *LSD1* was specifically linked to a decrease in E-cadherin expression [81,82,83]. E-cadherin is important for maintaining cell–cell adhesion among epithelial cells, which is a crucial factor in the initial stages of metastasis [145]. Reduced E-cadherin expression facilitates the detachment of cancer cells from the primary tumour mass, enabling them to transition to a more mesenchymal phenotype [145]. This transition promotes their ability to invade and migrate towards distant sites within the body [145]. However, it would be interesting to conduct in vivo experiments to better understand the association between KDM3A/LSD and CRC metastasis, adding further clarity to this relationship.

### 3.4. Understanding the Influence of Ubiquitination in CRC Metastasis

Ubiquitination is a form of histone modification that involves covalently attaching ubiquitin to a target protein and is mediated by the following three distinct classes of enzymes: the ubiquitin-activating enzyme (E1), ubiquitin-conjugating enzyme (E2), and ubiquitin-protein ligase (E3) [146]. In CRC, the dysregulation of multiple enzymes belonging to E2 and E3 classes has been linked to CRC metastasis [84,85,86]. The E2 enzyme family contains more than 40 members and regulates protein stability and ubiquitination [147]. E2 enzymes are important for tumour cell migration, invasion, cell cycle, proliferation, radiation, and drug resistance [147]. Among CRC patients with distant metastasis, it was observed that the Ubiquitin-Conjugating Enzyme E2 V1 (*Ube2v1*) and Ubiquitin-Conjugating Enzyme Variant 1a (*Uev1A*) (44/56 patients had exhibited high expression) were high in CRC metastasis and metastatic colon carcinoma, respectively [84,85,86]. Further, the investigation into the mechanisms involving these two genes revealed that *Ube2v1* facilitated the degradation of Sirtuin 1 (*Sirt1*) via Ubiquitin-Conjugating Enzyme 13 (*Ubc13*), which further led to a reduction in Histone H4 Lysine 16 Acetylation (*H4K16ac*) [84]. *Sirt1* and *H4K16ac* are important for autophagy regulation, where *Ubc13* acts as a modulator of the Ube2v1 function [84]. *Ube2v1* suppresses autophagy and promotes EMT and metastasis in CRC cell lines both in vitro and in vivo [84]. With regard to *Uev1A*, the upregulation of *Uev1A* led to an increased formation of the Uev1A-Ubc13 complex [85]. This subsequently promoted *NF-κB*, resulting in an increased Chemokine (C-X-C motif) Ligand 1 (*CXCL1*) expression, which further promotes metastasis [85].

**Figure 2:**
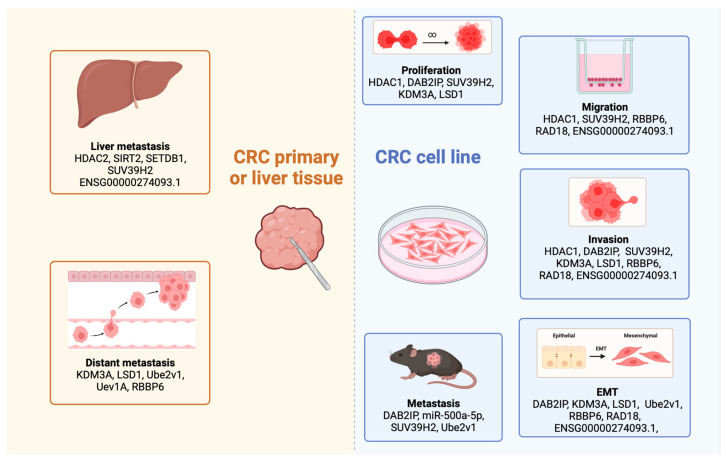
Summary of histone modifications linked with CRC metastasis in organ and cellular processes. Almost all histone modifications presented in this figure was derived from tissue analyses conducted on primary tumours, except for *SETDB1*, which specifically investigated liver metastatic lesions. Various CRC cell lines were used to show histone modifications and their correlation with cellular processes, including proliferation, migration, invasion, EMT, and metastasis. These include Histone Deacetylase 1 (*HDAC1*), Histone Deacetylase 2 (*HDAC2*), Sirtuin 2 (*SIRT2*), Disabled Homolog 2-Interacting Protein (*DAB2IP*), Suppressor of Variegation 3–9 Homologue 2 (*SUV39H2*), SET Domain Bifurcated Histone Lysine Methyltransferase 1 (*SETDB1*), Lysine-Specific Demethylase 3A (*KDM3A*), Lysine-Specific Histone Demethylase 1A (*LSD1*), Ubiquitin-Conjugating Enzyme E2 Variant 1 (*Ube2v1*), Ubiquitin-Conjugating Enzyme E2 variant 1 (*Uev1A*), Retinoblastoma-Binding Protein 6 (*RBBP6*).

E3 enzymes constitute one of the largest enzyme families and are involved in multiple functions. These functions include protein degradation, the mediation of protein-to-protein interactions, and activation of the inactivation of substrates [147,148]. Multiple E3 enzymes, such as Retinoblastoma-Binding Protein 6 (*RBBP6*) and *RAD18,* have been correlated with CRC metastasis [87,88,89]. These highly expressed E3 enzymes (*RBBP6* and *RAD18*) are associated with poor prognosis and possess the potential to be used as prognostic biomarkers [87,88,89]. Furthermore, in vitro, experimental results demonstrated that *RBBP6* and *RAD18* are involved in migration and invasion processes in CRC cell lines [88,89]. Moreover, E3 enzymes (*RBBP6* and *RAD18*) have been specifically linked to the EMT process, leading to the development of metastasis [88,89]. For example, the upregulation of *RBBP6* and *RAD18* (independently) increased the expression of N-cadherin and vimentin (which are EMT markers) while simultaneously reducing the expression of E-cadherin expression, which promotes EMT in vitro [88,89]. A closer examination of RBBP6’s role in the EMT process reveals how *RRBP6* activates the nuclear factor-κB via the ubiquitination of the inhibitor of nuclear factor-κB (*IκBα*), which promotes EMT and metastasis [88]. Understanding the signalling pathways regulated by these E3 enzymes can provide valuable insights into the mechanisms underlying CRC metastasis. A summary of the histone modification-based biomarkers in this section is outlined in Figure 2.

## 4. Conclusions

In this review, we assess the various DNA methylation and histone modification epigenetic changes associated with CRC metastasis. The identification and detection of epigenetic biomarkers in CRC metastasis pose several challenges, including the need for sensitivity and specificity to detect subtle changes in methylation or histone modifications unique to metastasis while ensuring accuracy. High-quality samples with a substantial tumour component and adequate quantity are essential to obtain accurate results. The absence of standardised methodologies for detecting and quantifying epigenetic modifications leads to inconsistencies across studies, making comparisons challenging. Additionally, certain detection techniques can be costly and require specialised equipment and expertise, hindering accessibility. Tumour heterogeneity further complicates matters, resulting in diverse epigenetic patterns within different areas of metastatic sites within the same patient. Moreover, the variability between patients presents challenges when attempting to identify consistent biomarkers.

Additional limitations arise from the predominant reliance on biopsied tissues for the prediction of CRC metastasis. These limitations include invasiveness, challenges in accessing tumour sites, the procedural risks associated with obtaining tissue biopsies for analysis, and a subsequent restriction in the frequency of sampling. The current focus is shifting towards liquid-based biopsy, which involves the analysis of biological fluids, such as blood, saliva, and urine. These have multiple advantages when compared to tissue biopsies, such as non-invasiveness, reduced health risks to patients during sample collection, and straightforward techniques to implement. Liquid biopsies consist of multiple components, such as circulating tumour DNA (ctDNA), circulating tumour cells (CTCs), exosomes, and miRNA. Liquid biopsies are emerging as promising alternatives to identify biomarkers for the detection and management of CRC metastasis, which could enhance patient care and clinical outcomes. While liquid biopsies provide a non-invasive route to acquire molecular insights in CRC metastasis, the constraints of single-omics analyses from these samples highlight the necessity for more comprehensive methodologies. The limitation of single-omics analyses is their restricted scope, focusing solely on one type of molecular data (e.g., genomics, transcriptomics, proteomics, or metabolomics). This approach might overlook complex interactions and pathways involving multiple biological layers, offering a limited understanding of CRC metastasis.

Integrated multiomics analyses, which combine data from multiple omics levels such as genomics, epigenomics, transcriptomics, proteomics, and metabolomics, enable a more thorough understanding of the complex biological mechanisms driving CRC metastasis. The application of deep learning and artificial intelligence analysis approaches further enhances the ability to detect and utilise novel markers of cancer metastasis [149]. This comprehensive approach helps identify interconnected molecular pathways, biomarkers, and potential therapeutic targets that would otherwise be missed when studying individual omics data. Moreover, the current research mainly focuses on the association between these markers and the presence of metastasis. However, further exploration into their mechanistic roles in the metastatic cascade is needed to identify potential therapeutic targets. Bridging these gaps is crucial for advancing the translation of these biomarkers from research settings to clinical practice, thereby enhancing the management and treatment outcomes for metastatic CRC.
